# Supplementary material for: Taxonomic identification, genomic analysis, and optimized chromium(VI) bioreduction by Microbacterium triticisoli sp. nov. M28T
Source: PeerJ. 2025 Oct 23;13:e20192. doi: 10.7717/peerj.20192 (PMC12554309; doi:10.7717/peerj.20192)
Supplement: Supplemental Information 1 [file peerj-13-20192-s001.docx]

**Methods for analysis phenotypic, physiological and biochemical characteristics, and chemical component of strain M28^T^**

Following inoculation on LB agar at 37°C for 3 days, colony morphology was observed. Fresh bacterial cells in the logarithmic growth phase were collected, washed sequentially with PBS buffer, fixed overnight, dehydrated through an ethanol gradient (30%, 50%, 70%, 80%, 90%, 95%, 100%), and vacuum-dried. Cellular morphology was examined using a scanning electron microscope (Quattro S FE-SEM, Japan). Gram staining was performed with a commercial kit (Guangdong Huankai Microbial Sci. & Tech. Co., Ltd.) according to the manufacturer's protocol. Growth characteristics were assessed in LB medium under varying conditions: Temperature range: 15-42°C (tested at 15, 20, 25, 28, 30, 35, 37, 40, 42°C), pH gradient: 4.0-11.0 (0.5 increments, adjusted with 1 M NaOH/HCl)，NaCl concentrations: 0-15% (w/v).

Physiological characterization was performed following standard protocols. Biochemical tests such as Voges-Proskauer (acetoin detection) and methyl red (acid production) assays, catalase activity, oxidase activity, and substrate utilization profiles (including cellulose hydrolysis, gelatin liquefaction, starch degradation, Tween 40 hydrolysis, milk coagulation and peptonization, urea hydrolysis, hydrogen sulfide production, and nitrate reduction) were assessed according to the literature (Li et al., 2016; Gonzalez et al., 1978). Carbon substrate utilization was evaluated using Biolog GEN III MicroPlates™. Additionally, carbohydrate fermentation profiles and enzymatic activities were determined with API 50CH and API ZYM systems following manufacturers' protocols.

Log-phase cells were harvested for chemotaxonomic characterization，including fatty acid composition, polar lipid profiling, and respiratory quinone analysis (Uvarova et al., 2020). Fatty acid methyl esters (FAMEs) were prepared through saponification-methylation derivatization and subsequently analyzed using the Sherlock® Microbial Identification System (MIDI Inc., Newark, DE) (Sasser et al., 1990). Polar lipid extraction involved two-dimensional thin-layer chromatography on silica gel H plates, with specific detection reagents: phosphomolybdic acid for total lipids, molybdenum blue for phospholipids, and α-naphthol for glycolipids (Minnikin et al., 1977). Respiratory quinones were isolated via preparative Thin-layer chromatography (TLC) (Cai, 2014), followed by reversed-phase HPLC analysis using an Agilent 1260 Infinity system equipped with a C18 column (4.6 × 250 mm, 5 μm particle size).

**Results of phenotypic and physiological and biochemical characteristics**

Phenotypic characterization of strain M28^T^ revealed circular, convex, light yellow colonies (1-2 mm diameter) on LB agar after 72 h at 30 ^o^C (Figure 1a). Scanning electron microscopy (SEM) demonstrated short rod-shaped cells (0.5-1.0 × 0.8-2.0 μm) (Figure 1b). Physiological profiling showed positive reactions for catalase, urease, Tween 40 hydrolysis, gelatin liquefaction, and nitrate reduction, but negative results for starch hydrolysis, cellulase activity, methyl red, Voges-Proskauer, and peptonization. The strain exhibited mesophilic growth between 15-38 °C (optimum 25-30 °C), neutrophilic pH tolerance (5.5-10.0; optimum pH 7.0), and halotolerance up to 8% NaCl (w/v; optimum 1%). No growth was observed under anaerobic conditions in the culture medium using the liquid paraffin cover method, confirming obligate aerobic metabolism. Comparison of phenotypic and biochemical characteristics of strain M28^T^ with closely related *Microbacterium* type strains are summarized in Table 1 and Table 2.

The carbon source utilization, acid production and enzymatic properties of strain M28^T^ and related type strains were investigated using the GEN III MicroPlate™, API 50CH and API ZYM systems. In the GEN III test, strain M28^T^ exhibited unique carbon source utilization patterns, characterized by efficient metabolism of multiple carbon sources such as dextrin, D-maltose, D-trehalose and gentiobiose. While strain M28^T^ shared utilization of certain carbon sources with *M. aquimaris* JCM 15625^T^, significant metabolic divergence was evident through differential substrate assimilation patterns (Table 3). The Biolog similarity index between strain M28^T^ and *M. aquimaris* JCM 15625^T^ was 70.9%, significantly below the 95% species delineation threshold, confirming significant phenotypic divergence.

Enzymatic profiling using the API ZYM® kit (Humble et al., 1997) revealed strain M28^T^ shared conserved enzymatic activities with *M. aquimaris* JCM 15625^T^: exhibiting positive reactions for esterase (C4), leucine arylaminase, naphthol-AS-Bl-phosphate hydrolase, and α-glucosidase, while consistently lacking lipoid lipase (C14), trypsin, tryptic curdling protease, and β-fucosidase activity. Notably, significant divergences were observed in 11 additional hydrolase activities compared to type strains, with comprehensive data detailed in Table 4. The API 50CH^®^ assay was conducted to delineate carbohydrate acidification patterns (Johansson et al., 1995). Strain M28^T^ and related type strains shared acid production from core substrates (e.g., galactose, sucrose) but failed to acidify carbohydrates like adonitol. The distinct substrate utilization data for strain M28^T^ and related type strains were provided in Table 4.

**Results of chemical taxonomy**

Chemotaxonomic analysis revealed that strain M28^T^ had iso-C15:0, pre-C15:0, pre-C16:0, and pre-C17:0 as the major fatty acids (Table 5). While sharing the principal fatty acid classes with *Microbacterium* type strains, quantitative divergence was observed.The polar lipids pattern of strain M28^T^ consisted of phosphatidylglycerol (PG), diphosphatidylglycerol (DPG), and an unknown glycolipid (GL) (Figure 2), which was similar to that of the type strain. Respiratory quinones were predominantly distributed as MK-10, MK-11, and MK-12.

**References**

Cai, L. 2014. Thin layer chromatography. Curr. Protoc. Essent. Lab. Tech. 8(1), 6-3. <https://doi.org/10.1002/9780470089941.et0603s08>

Gonzalez, C., Gutierrez, C., Ramirez, C., 1978. Halobacterium vallismortis sp. nov. An amylolytic and carbohydrate-metabolizing, extremely halophilic bacterium. Can. J. Microbiol. 24(6), 710-715. <https://doi.org/10.1139/m78-119>

Humble, M. W., King, A., Phillips, I. 1977. API ZYM: a simple rapid system for the detection of bacterial enzymes. J. Clin. Pathol. 30(3), 275-277.https://doi.org/10.1136/jcp.30.3.275

Johansson, M. L., Sanni, A., Lönner, C., Molin, G. 1995. Phenotypically based taxonomy using API 50CH of lactobacilli from Nigerian ogi, and the occurrence of starch fermenting strains. Int. J. Food Microbiol. 25(2), 159-168. [https://doi.org/10.1016/0168-1605(94)00096-O](https://doi.org/10.1016/0168-1605(94)00096-O)

Li, Q., Chen, X., Jiang, Y., Jiang, C. 2016. Cultural, Physiological, and Biochemical Identification of. Actinobacteria: basics and biotechnological applications, 87. <http://dx.doi.org/10.5772/61462>

Minnikin, D.E., Patel, P.V., Alshamaony, L., Goodfellow, M., 1977. Polar lipid composition in the classification of Nocardia and related bacteria. Int. J. Syst. Evol. Microbiol. 27(2), 104-117. https://doi.org/10.1099/00207713-27-2-104

Sasser, M., 1990. Identification of bacteria by gas chromatography of cellular fatty acids. MIDI Technical Note 101. 101-107. https://doi.org/10.1099/IJSEM.0.003038

Uvarova, Y., Bryanskaya, A. V., Rozanov, A. S., Shlyakhtun, V. N., Demidov, E. A., Starostin, K. V., Peltek, S. E., 2020. An integrated method for taxonomic identification of microorganisms. Vavilov J. Genet. Breed. 24(4), 376. <https://doi.org/10.18699/VJ20.630>


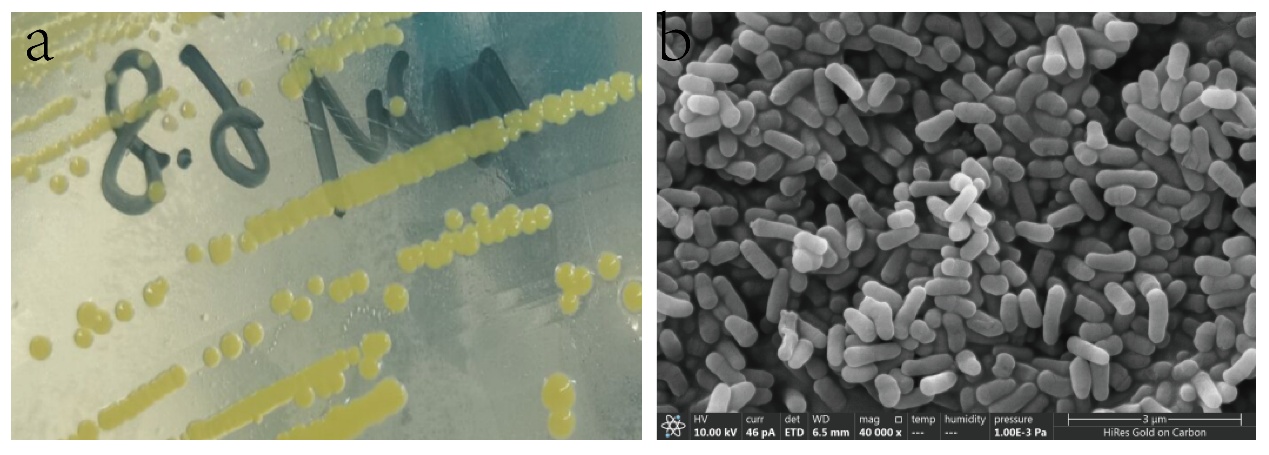


Figure 1 (a) Colony of strain M28 on LB agar plate; (b) Morphology of strain M28 cells under scanning electron microscopy.


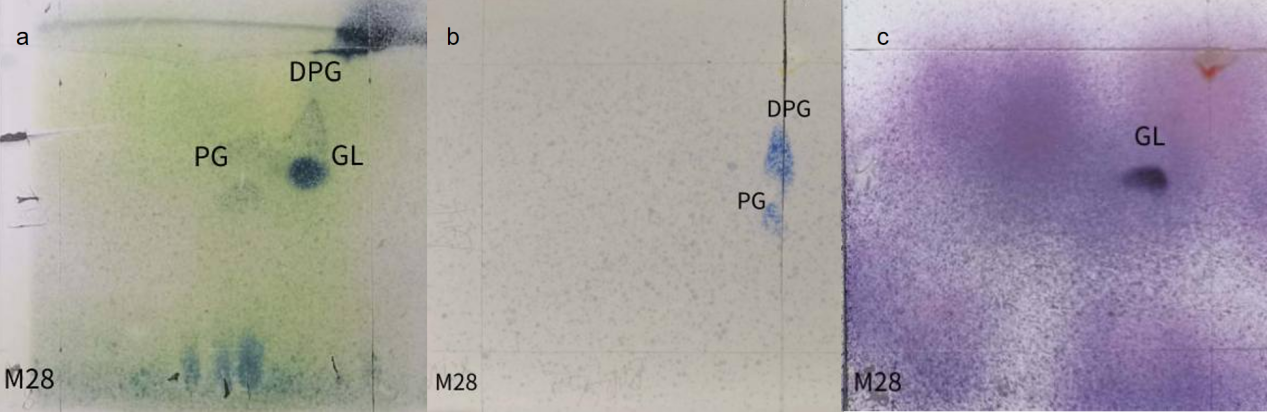


Figure 2 Two-dimensional thin layer chromatographic analysis of polar lipids of strain M28^T^; (a) total lipids, (b) phospholipids, (c) aminolipids. PG: phosphatidylglycerol, DPG: diphosphatidylglycerol, GL: an unknown glycolipid.

Table 1 Differential characterization of strain M28^T^ with related type strains of *Microbacterium*. Strains: 1, strain M28^T^; 2, *M. profundi* Shh49^T^; 3, *M. murale* CCM 7640^T^, 4, *M. aquimaris* DSM 19713^T^. +, positive; –, negative; ND, not detected.

| **Characteristic** | **1** | **2** | **3** | **4** |
| --- | --- | --- | --- | --- |
| Colour of colonies | Light yellow | Yellow | Yellow | Yellow |
| Motility | - | - | - | - |
| Morphology | short rod | short rod | short rod | short rod |
| Length | 0.8-2.0 | 1.0-2 | 2-3 | 1.0-1.5 |
| Width | 0.5-1.0 | 0.4-0.6 | 1-1.2 | 0.6-0.8 |
| 37 ℃ | + | - | - | + |
| pH range (optimal) | 5.5-10(7.0) | 6.0-9.5(7.0-8.0) | ND | 6.0-10.0(7.0-8.0) |
| DNA G+C content (mol%) | 67.76 | 66.8 | ND | 69.3 |
| Major fatty acids | iso-C_15:0_, anteisoC_15:0_, isoC_16:0_, isoC_17:0_, anteisoC_17:0_ | iso-C_15:0,_ anteisoC_15:0_, isoC_16:0_, anteisoC_17：0_ | anteisoC_15:0_, isoC_16:0_, anteisoC_17:0_ | anteisoC_15:0_, isoC_16:0_, anteisoC_17:0_ |

Table 2 Enzymatic characteristics experiments of strain M28^T^, *M. aquimaris* DSM 19713^T^ and *M. profundi* Shh49^T^. +, positive; –, negative.

|  | **M28^T^** | ***M. aquimaris* DSM 19713^T^** | ***M. profundi* Shh49^T^** |
| --- | --- | --- | --- |
| Oxidase test | - | - | - |
| Peroxidase experiment | + | + | + |
| Urease test | - | - | - |
| Lipase experiment | - | - | - |
| Gelatin liquefaction | + | + | + |
| Milk coagulation and peptization | - | - | - |
| starch hydrolysis | - | - | - |
| Cellulose decomposition | - | - | - |
| Nitrate reduction | + | + | - |
| MR experiment | - | - | - |
| V-P experiment | - | - | - |
| Indole test | - | - | - |
| hydrogen sulfide | - | - | - |

Table 3 Results of carbon source utilization by strain M28^T^ and related type strains.

| **Carbon source utilization type** | **M28^T^** | ***M. aquimaris* DSM 19713^T^** | ***M. profundi* Shh49^T^** | ***M. murale* CCM 7640^T^** |
| --- | --- | --- | --- | --- |
| **The available carbon source** | Dextrin, D-maltose, D-trehalose, gentiobiose, sucrose, turanose, stachyose, melezitose, melibiose, L-glutamic acid, p-hydroxy-phenylacetic acid D-cellobiose, α-D-glucose D-mannose, D-fructose, D-galactose, L-rhamnose, D-mannitol, pectin, acetic acid | D-cellobiose, α-D-glucose D-mannose, D-fructose, D-galactose, L-rhamnose, D-mannitol, pectin, acetic acid, D-salicin, D-gluconic acid, L-lactic acid, acetoacetic acid,  D-lyxose, Melezitose，melibiose, raffinose，xylose，5-Ketogluconate | acetate, L-arabinose, L-arginine, L-aspartate, cellobiose, D-fructose, fumarate, D-galactose, D-glucose, glycerol, isoleucine, lactate, lactose, lysine, malate, maltose, D-mannitol, D-mannose, propionate, pyruvate, raffinose, rhamnose, ribose, salicin, L-serine, succinate, sucrose, trehalose, L-valine, xylose | N-acetyl-D-galactosamine, N-acetyl-D-glucosamine, cellobiose, D-galactose, D-glucose, D-mannose, maltose, D-ribose, sucrose and trehalose, hydrolysed aesculin, pNP-a-D-glucopyranoside, pNP-b-D-glucopyranoside, bis-pNP-phosphate, pNP-phenylphosphonate |
| **The carbon source that can be utilized in a weak and trace amount** | D-salicin, N-acetyl-D-glucosamine, N-acetyl-D-galactosamine, inosine, aminoacetyl-L-proline, L-aspartic acid, D-gluconic acid, L-lactic acid, α-hydroxy-butyric acid, α-keto-butyric acid, α-D-lactose, glycerol, propionic acid | Dextrin, D-maltose, D-trehalose, gentiobiose, sucrose, D-turanose, β-formyl-D-glucoside, L-fructose, glycerol, gelatin, L-alanine, L-glutamic acid, glucosylamine, citric acid, Tween 40, β-hydroxy-D, L-butyric acid, propionic acid, α-D-lactose, glycerol, propionic acid | N-acetylglucosamine, adipic acid, L-alanine, capric acid, citrate, L-cysteine, ethanol, formate, glycine, L-histidine, myo-inositol, malonate, L-methionine, potassium gluconate, phenylacetic acid, sorbitol, sorbose, trisodium citrate | D-adonitol, myo-inositol,  trans-aconitate, adipate, 4-aminobutyrate, azelate, citrate, itaconate,  mesaconate, suberate, L-tryptophan, DL-3-hydroxybenzoate, DL-4-hydroxybenzoate. |

Table 4 Results of API ZYM and API 50CH testes of strain M28^T^ and related type strain. +, positive; –, negative; w, weakly positive. 1, M28^T^；2, *M. aquimaris* DSM 19713^T^；3, *M. resistens* NBRC103078^T [1]^; 4, *M. testaceum* NBRC12675^T^ ^[2]^; 5, *M. profundi* Shh49^T^ ^[3]^.

| **project** | **1** | **2** | **3^*^** | **4^*^** | **5*** |
| --- | --- | --- | --- | --- | --- |
| Enzyme activities (API ZYM) |  |  |  |  |  |
| alkaline phosphatase | + | - | - | - | - |
| Lipoprotein lipase (C8) | w | w | + | + | w |
| Valine arylamine enzyme | w | + | + | + | + |
| cystine arylamidase | - | - | + | + | - |
| acid phosphatase | + | w | + | + | - |
| alpha-galactosidase | w | w | - | w | - |
| β-galactosidase | + | - | + | + | + |
| β-glucuronidase | - | - | + | + | + |
| β-glucosidase | + | + | - | - | + |
| N-acetyl-β-glucosaminidase | + | + | + | - | - |
| alpha-mannosidase | + | - | + | + | + |
| alpha-galactosidase | + | - | - | - | - |
| Acid production from (API 50CHB) |  |  |  |  |  |
| alpha-galactosidase | + | + | + | - | + |
| D-arabinose | + | - | - | - | + |
| L-arabinose | + | - | - | + | + |
| ribose | + | + | + | - | - |
| D-xylose | + | + | - | + | - |
| β-methyl-D-xyloside | + | + | - | - | + |
| sorbose | - | - | - | + | - |
| Mannitol | + | + | - | + | + |
| α-methyl-D-mannoside | + | - | - | + | + |
| α-methyl-D-glucoside | + | - | - | + | + |
| N-acetyl-glucosamine | - | - | + | + | - |
| Amygdalin | w | + | + | + | - |
| arbutin | w | + | - | + | + |
| saligenin | w | + | + | + | - |
| lactose | + | + | + | - | + |
| melibiose | w | w | - | - | - |
| Trehalose | + | - | + | + | + |
| melitriose | + | + | - | + | - |
| raffinose | w | - | - | - | - |
| starch | w | - | - | + | - |
| glycogen | w | - | - | + | - |
| xylitol | - | - | + | + | - |
| gentiobiose | + | w | - | - | - |
| D-turanose | + | w | + | + | - |
| D-lyxose | - | - | + | + | - |
| D-tagatose | - | - | - | + | - |
| L-fucose | + | + | - | - | + |
| gluconate | - | - | + | + | - |
| 2-keto-gluconate | - | - | + | - | - |
| 5-keto-gluconate | - | - | + | - | - |

[1] Funke G, Lawson P A, Nolte F S, et al. *Aureobacterium resistens* sp. nov., exhibiting vancomycin resistance and teicoplanin susceptibility[J]. FEMS Microbiology Letters, 1998, 158(1): 89-93.

[2] Zhang Y, Ren H, G. Z. *Microbacterium hydrothermale* sp. nov., an actinobacterium isolated from hydrothermal sediment[J]. International Journal of Systematic and Evolutionary Microbiology, 2014, 64(Pt 10): 3508-3512.

[3] Wu Y H, Wu M, Wang C S, et al. *Microbacterium profundi* sp. nov., isolated from deep-sea sediment of polymetallic nodule environments[J]. International journal of systematic and evolutionary microbiology, 2008, 58(12): 2930-2934.

Table 5 Fatty acid composition of strain M28^T^ and the related type strains.

| **Peak Name** | **M28^T^** | ***M.murale* CCM 7640^T^** | ***M. aquimaris* DSM 19713^T^** |  |
| --- | --- | --- | --- | --- |
| iso C_14:0_ | 2.32 | 1.6 | 0.7 |  |
| C_14:0_ | 0.12 | 0.6 | 0.11 |  |
| iso C_15:0_ | 11.67 | 4.9 | 15.77 |  |
| anteiso C_15:0_ | 35.60 | 48.5 | 33.05 |  |
| iso C_16:0_ | 28.44 | 20.1 | 8.76 |  |
| C_16:0_ | 1.19 | 2.9 | 0.99 |  |
| iso C_17:0_ | 4.96 | 1.0 | 10.37 |  |
| anteiso C_17:0_ | 14.77 | 20.0 | 29.55 |  |
