# Supplementary material for: Taxonomic identification, genomic analysis, and optimized chromium(VI) bioreduction by Microbacterium triticisoli sp. nov. M28T
Source: PeerJ. 2025 Oct 23;13:e20192. doi: 10.7717/peerj.20192 (PMC12554309; doi:10.7717/peerj.20192)
Supplement: Supplemental Information 2 [file peerj-13-20192-s002.docx]

Figure S1 Neighbor-joining phylogenetic tree based on 16S rRNA gene sequences, showing the phylogenetic position of strain M28^T^ among related strains.

*Microbacterium keratanolyticum* DSM 8606 ^T^

*Microbacterium phyllosphaerae* DSM 13468 ^T^

*Microbacterium profundi* Shh49^T^

*Microbacterium murale* CCM 7640^T^

*Microbacterium diaminobutyricum* KU843548 ^T^

*Microbacterium pullorum* Sa4CUA7^T^

*Microbacterium oleivorans* NBRC 103075^T^

*Microbacterium paraoxydans* DSM 15019^T^

*Microbacterium algeriense* G1 DSM 109018^T^

*Microbacterium saperdae* DSM 20169^T^

*Microbacterium oxydans* DSM 2057 ^T^

*Microbacterium maritypicum* AJ85391 ^T^

*Microbacterium azadirachtae* DSM 23848^T^

*Microbacterium arabinogalactanolyticum* JCM 9171^T^

*Microbacterium resistens* NBRC 103078^T^

*Microbacterium testaceum* NBRC 12675^T^

***Microbacterium triticisoli* M28**^T^

*Microbacterium aquimaris* DSM 19713^T^

*Microbacterium allomyrinae* NBRC 115127^T^

*Microbacterium ureisolvens* DSM 103157^T^

*Microbacterium aureliae* KF793922^T^

*Microbacterium sulfonylureivorans* LAM7116^T^

*Microbacterium fluvii* AB286028^T^

*Microbacterium binotii* JCM 16365^T^

*Microbacterium neimengense* JN408293^T^

*Agromyces aureus strain* AR33^T^

99

83

87

53

97

63

85

64

64

73

70

0.0050
