# Supplementary material for: Taxonomic identification, genomic analysis, and optimized chromium(VI) bioreduction by Microbacterium triticisoli sp. nov. M28T
Source: PeerJ. 2025 Oct 23;13:e20192. doi: 10.7717/peerj.20192 (PMC12554309; doi:10.7717/peerj.20192)
Supplement: Supplemental Information 3 [file peerj-13-20192-s003.docx]

Table S1 Heavy metal related genes in strain M28^T^

| Metal | Resistant genes | Gene ID | Description |
| --- | --- | --- | --- |
| Cu | copA | gene0042 | P-type Cu^+^ transporter [EC:7.2.2.8] |
|  | copB | gene0044 | P-type Cu^2+^ transporter [EC:7.2.2.9] |
|  | cutC | gene0431 | copper homeostasis protein |
|  | copC | gene2009 | copper resistance protein C |
|  | actP | gene0898 | cation/acetate symporter |
| As | arsR | gene2894 | ArsR family transcriptional regulator,  arsenate/arsenite/antimonite-responsive transcriptional repressor |
|  | arsB | gene2896、2897 | arsenite transporter |
| Mo | modB | gene1952 | molybdate transport system permease protein |
|  | modC | gene1953 | molybdate transport system ATP-binding protein [EC:7.3.2.5] |
| Mg | corA | gene1207 | magnesium transporter |
| Mn | mntc | gene3157 | manganese transport system substrate-binding protein |
|  | mntA | gene3158 | manganese transport system ATP-binding protein [EC:7.2.2.5] |
|  | mntB | gene3159 | manganese transport system permease protein |
| Cd | czcD | gene2139 | cobalt-zinc-cadmium efflux system protein |
