# Supplementary material for: Taxonomic identification, genomic analysis, and optimized chromium(VI) bioreduction by Microbacterium triticisoli sp. nov. M28T
Source: PeerJ. 2025 Oct 23;13:e20192. doi: 10.7717/peerj.20192 (PMC12554309; doi:10.7717/peerj.20192)
Supplement: Supplemental Information 4 [file peerj-13-20192-s004.docx]

Table S2 Genes related to lignocellulose degradation annotated in M28^T^

| Gene ID | Family Description |
| --- | --- |
| gene 3034 | acetyl xylan esterase（EC3.1.1.72）； |
| gene 0126 | cellobiose dehydrogenase (EC 1.1.99.18); |
| gene 2341 | vanillyl-alcohol oxidase (EC 1.1.3.38) |
| gene 0337 | endo-1,4-beta-xylanase (EC 3.2.1.8)  beta-xylosidase (EC 3.2.1.37) |
| gene 0169 | endo-1,3-beta-xylanase (EC 3.2.1.32); |
| gene 0343 | beta-mannosidase（EC3.2.1.25） |
| gene0457 | endoglucanase (EC 3.2.1.4) |
| gene 1140 | manganese peroxidase (EC 1.11.1.13);  versatile peroxidase (EC 1.11.1.16);  lignin peroxidase (EC 1.11.1.14);  peroxidase (EC 1.11.1.-) |
