# Supplementary material for: Taxonomic identification, genomic analysis, and optimized chromium(VI) bioreduction by Microbacterium triticisoli sp. nov. M28T
Source: PeerJ. 2025 Oct 23;13:e20192. doi: 10.7717/peerj.20192 (PMC12554309; doi:10.7717/peerj.20192)
Supplement: Supplemental Information 5 [file peerj-13-20192-s005.pdf]

中 国 典 型 培 养 物 保 藏 中 心
CHINA CENTER FOR TYPE CULTURE COLLECTION (CCTCC)
Tel: (027) 6875 2319 Fax: (027) 6875 4833 E-mail:cctcc@whu.edu.cn Wuhan University, Wuhan 430072, P. R. China

STATEMENT IN THE CASE OF AN ORIGINAL DEPOSIT

I. IDENTIFICATION OF THE MICROORGANISM

Identification reference given by
DEPOSITOR: **Xiufeng Long**
Microbacterium triticisoli **sp. nov.**
Strain: M28 T

Accession number given by the
CCTCC AA 2022021T

II. SCIENTIFIC DESCRIPTION AND/OR PROPOSED TAXONOMIC DESIGNATION

The microorganism identified was accompanied by:

■ A scientific description

■ A proposed taxonomic designation

(Mark with a black where applicable)

III. RECEPT AND ACCEPTANCE

This International Depositary Authority accepts the microorganism identified, which was received by it on August 10, 2022.

This strain has been checked for viability in CCTCC and was stored by standard methods used in CCTCC. The strain has been deposited and will be availability without restrictions from the CCTCC in accordance with the Rules of the Bacteriological Code (1990 revision) are revised by the ICSP at the plenary sessions in Sydney and Paris.

IV. INTERNATIONAL DEPOSITARY AUTHORITY

Signature of person to represent the International Depositary Authority:

Name: Fang Peng Date: June 25, 2025

中国典型培养物保藏中心 业务专用章

CHINA CENTER FOR TYPE CULTURE COLLECTION (CCTCC)
WUHAN UNIVERSITY, WUHAN 430072, P. R. CHINA
Tel: +86-(027)-6875 2319 Fax: +86-(027)-6875 4833
E-mail: cctcc@whu.edu.cn
