# Supplementary material for: Taxonomic identification, genomic analysis, and optimized chromium(VI) bioreduction by Microbacterium triticisoli sp. nov. M28T
Source: PeerJ. 2025 Oct 23;13:e20192. doi: 10.7717/peerj.20192 (PMC12554309; doi:10.7717/peerj.20192)
Supplement: Supplemental Information 6 [file peerj-13-20192-s006.pdf]

JAPAN COLLECTION OF MICROORGANISMS

Microbe Division, RIKEN BioResource Research Center
3-1-1 Koyadai, Tsukuba, Ibaraki 305-0074, Japan

Phone : +81 29 836 9556
Fax : +81 29 836 9561
E-mail : inquiry.jcm@riken.jp

December 9, 2022

Dr. Xiufeng Long
College of Biological and Chemical Engineering,
Guangxi University of Science and Technology
268 Avenue Donghuan, Chengzhong District, Liuzhou, Guangxi
People's Republic of China

Dear Dr. Long,

The microorganisms that you deposited have been accessioned with the following JCM numbers:

JCM 35796 *Microbacterium triticisoli*
<- X. Long; Guangxi Univ. of Sci. & Technol., China; M28

JCM 35797 *Rhodococcus ferula*
<- X. Long; Guangxi Univ. of Sci. & Technol., China; Z13

When you make contact with JCM on this matter, please refer to the JCM numbers.

Thank you very much for your cooperation.

Yours sincerely,

Takao Iino, Ph.D.
Curator

Moriya Ohkuma, Ph.D.
Director
Microbe Division / Japan Collection of Microorganisms
RIKEN BioResource Research Center

NOTICE: *This document is* ***not*** *a certificate of deposition and availability of the microorganism(s) deposited, which is necessary for the purpose of valid publication of a new scientific name in the International Journal of Systematic and Evolutionary Microbiology (IJSEM). If you require the certificate, please contact us.*

国立研究開発法人 理化学研究所 バイオリソース研究センター 微生物材料開発室
〒305-0074 茨城県つくば市高野台3丁目1番地1
